# Progress in Medicine

**Published:** 1896-08-15

**Authors:** 


### Progress in Medicine.

### TYPHOID FEVER.

The immense importance of a test by which typhoid can be quickly and certainly recognised, and the colon bacillus separated from Eberth's, is shown by the discussion on Elsner's[1] test and the so-called serum diagnosis. In the former ordinary gelatin is prepared with an infusion of potatoes (500 grams to a litre of water). To this is added enough normal sodium solution to give a slight acid reaction. After filtering and sterilizing one per cent. of potassium iodide is added. If this is inoculated from fæces the growth of all microbes is found to be prevented except the colon and Eberth's bacillus. Colonies of the latter appear in forty-eight hours as small, shining clear drops, while those of the latter are larger, brownish, and coarsely granular. Elsner thus isolated the bacilli in fifteen out of seventeen cases of typhoid, the two where it failed being convalescent ones. Chantemesse and many other have confirmed these results, showing that it is a test of great value, even in the earliest stages, and that by it we can decide when a patient ceases to be infectious with some degree of certainty.

To apply the serum test Widal[2] obtains blood with aseptic precautions from a typhoid patient, separates the serum, and introduces a few drops of the latter into a tube containing ten or fifteen times as much broth inoculated with a culture of Eberth's bacillus, which is then placed in the incubator at a temperature of 39 deg. for twenty-four hours. The broth is then found to be cloudy, having white flakes at the bottom, the precipitate being composed of masses of bacilli, which are seen under the microscope to be deprived of motion. In ordinary typhoid cultures there is a uniform cloud and a "watered silk" appearance, but the fine dust-like particles are absent, and the bacilli are separate and mobile. Widal and Dieulafoy[3] have tested the blood of many other persons in health or suffering from various diseases, and found no such immobilisation and agglutination of bacteria on the addition of their serum. Where bacteriological methods are practicable either of these tests may be of use. Bouy[4] points out that in animals an injection containing the bacillus is much increased in virulence if the toxin of the colon bacillus be added. Again, Klein has in his recent lectures discussed in full detail the differences between the two bacilli, to which we shall refer later on. Among other writers on the same subject is D. A. Welsh,[5] who mentions that the addition of 1 in 7,000 of formalin will inhibit the growth of the typhoid bacillus, while it has no effect on the B. coli. Stern[6] gives evidence to show that high febrile temperature has no inhibitory action on the typhoid organism, and that the serum of convalescent patients varies so much in its protective power that it is only a few cases which can yield a serum of therapeutic value.

The question whether London typhoid can be produced by the enormously diluted poison contained in the great mass of Thames water receives light not only from the Tees Valley epidemic, but also from the Merrimac one. Here two outbreaks arose from contamination of a large river nine miles above the affected town, and a third where the town was twenty miles from the contamination. Shirley Murphy[7] pointed out in connection with this topic that an excess of typhoid in London over a large area coincided with a flood in the Thames and Lea, which excess did not occur in districts taking their supply from other places. The difficulty of the diagnosis of the bacillus is shown by McCrone's[8] examination of some oysters, where he found a non-pathogenic bacillus very like the typhoid, but growing preferably at normal room temperatures. Similar pseudotyphoid bacilli have been described by Sanarelli and Cassedebat.

**Treatment.**—A. H. W. Clemow[9] describes a case where a temperature of 107 deg. with a dilated right heart was treated successfully by venesection. Possibly some pulmonary complication was relieved by the bleeding and the hyperpyrexia indirectly. Benario[10] produced a fall of 1 to 2·6 deg. by the use of citrophen without any ill effect except profuse sweating. Schilling[11] advocates injections of 15 to 45 drops of a 10 per cent. solution of camphorated oil for collapse. When 7 to 15 grains of camphor have thus been injected great relief is obtained. Bignami[12] has treated 200 patients with a mortality of only 3 per cent. by phenacetin. He gives 7½ grain doses every four hours for the first week, and afterwards every six hours till the temperature is normal through two entire days. H. McCormick[13] has given guiacol externally to 45 typhoid patients in quantities of from 2 to 25 drops. This was rubbed in over the right iliac region and then covered with oil silk. With ordinary care and moderate doses no depressing effects ensue. One patient had as many as 78 applications. The antithermic effect is not produced by internal administration. Barth and Stoltzenburg[14], however, met with so much collapse and sweating, that they discarded the use of guiacol and creosote. Applications of cocaine failed in their hands to produce any fall of temperature, and they believe the action of the former substances to be confined to irritation of the sweat glands, which prevents the accumulation of heat.

R. M. Simon[15] believes that typhoid mortality is being lessened generally by improved treatment. Antiseptic remedies are useful in preventing secondary infection. He prefers fifteen drops of oil of turpentine with one drop of liquor potassæ in mucilage every four hours, though Short gives instances where it had untoward effects. Simon, moreover, holds that with the same constant care as used in the Brandt treatment, equally good results can be got without baths. In the first week a single dose of castor oil may be given to check diarrhœa, but in the later stages he advises bismuth, or starch and opium enemata. If the abdomen is convex we must use the greatest caution in allowing peristaltic action to go on, otherwise constipation for more than 48 hours must be prevented. He advocates the administration of opium when the nearness of perforation is shown by great distension and tremor. The drug, too, is invaluable when fretfulness and mental anxiety are present. As to diet, he prefers peptonised milk alone every three hours, the quantity not to exceed 2½ pints daily. In a discussion which followed his paper, Saundby objected to the Brandt system because most cases came in too late, and he has had equally good results without it in later years. Foxwell spoke, however, in favour of the tepid bath plan, and instanced Barr's death-rate of only 4 per cent. He preferred as antiseptics calomel or corrosive sublimate, with opium if necessary. Stacey Wilson advised that only half the milk should be peptonised, and Short advocated the Woodridge treatment by constant draughts of sterilized water and minute doses of calomel and podophyllin. Barrs, of Leeds,[16] would relax the present strictness of diet, not only in convalescence but in some cases during the acute stage. Meat and even fruit are given by him to avoid the harm which he thinks is done to the patient's general health by a prolonged liquid diet. His views have, however, naturally met with much opposition.

A great deal of work has been done on serum therapy. Pfeiffer and Kolle[17] find that the serum of typhoid patients in the first week of convalescence has from 20 to 100 times the destructive power of normal serum on typhoid bacilli. Of even higher strength was the serum of goats which had been immunised by increasing doses of bacilli, yet the serum does not destroy the bacilli *in vitro*, nor is it directly antitoxic when tested with the typhotoxin, nor, again, does it destroy the bacilli by increased phagocytosis. Beumer and Peiper[18] have employed with scanty results serum from inoculated sheep. At present it seems that the treatment must be very early if it is to be of use. Rumpf[19] has treated patients by injections of dead cultures of B. pyocyaneus, and reports some success. Considerable variety of opinion exists on the causation and frequency of laryngeal ulcers in typhoid. Kanthack and Drysdale[20] find in recent records at St. Bartholomew's that it occurred in 26 per cent. of the cases. The authors believe it is caused by pyogenetic cocci, while others refer the affection to the typhoid microbe. Ulcers on the soft palate and tongue occur in about a sixth of all cases according to Devic.[21] The mouth should be kept clean, but caustic is not required. Their progress affords, he thinks, a valuable indication of the condition of the intestinal ulcers, and if they heal when the temperature falls no relapse is to be expected. Svehla[22] found the bacillus in a case of apyrexial fever, and noticed in it also the diazo reaction. This reaction he obtained, too, in a culture of Eberth's bacillus, while one of B. Coli failed to give it. These points are of value in clearing up doubts as to the nature of apyrexial fever and of the diazo test. Hengst[23] collected 1,228 typhoid cases and found that 28 instances of otitis media were present among them, which amounts to about 2½ per cent. The affection appears about the third week, and nearly always terminates favourably. It does not seem to follow the use of quinine, but may be induced by a draught or carelessness in bathing. He recommends a leech and warm boracic instillations, and, if necessary, incision of the membrane. Lannois[24] records a case of the very rare abscess of the liver following pylephlebitis. There were a number of small abscesses, and the bacillus was found in the pus. His case illustrates the rule of Sir Dyce Duckworth, that the sequels of enteric are met with when convalescence is more or less established, though the latter denies that suppuration results from post-enteric phlebitis.[25] Among other sequelæ noticed by Duckworth are attacks of silence lasting for weeks, and often following otitis or deafness. This is not a true aphasia, and may be accompanied with stupor and paresis. A large variety of brain and spinal lesions are on record, and enteric seems to be followed by neuritis oftener than any other infectious fever except diphtheria and influenza. A useful summary of the better known sequelæ and their mode of origin is given in this author's "Lumleian Lectures." Hare[26] writes in favour of the view that moderate pyrexia is Nature's means of combating toxins, and that the benefit of cold baths lies not in the abstraction of heat, but in the increased metabolism and the rapidity of all vital processes which they cause. In support of this view, and the harmfulness of antipyretic drugs, he collects a large amount of recent experimental evidence. T. Lovett[27] discusses the course of typhoid in children and finds the mortality is about half that of adults, while the course is shorter, lasting a little less than three weeks. Many children under ten reach the end of the pyrexia before ten days. Constipation is more frequent than diarrhœa, while hæmorrhage and perforation are very uncommon before ten years. Patients aged only fifteen and eighteen months are reported by W. O. Bridges.[28] The youngest showed distension of the abdomen, diarrhœa rose spots, and the diazo reaction. Much benefit was observed from the use of the cold water coil placed on the abdomen when the temperature exceeded 102 degrees. A discussion took place at the Royal Medical Chirurgical Society on Malta fever, where L. Hughes noticed the relapsing character of the fever, its very long duration, and the subsequent anæmia. Inoculation of the micrococcus produced a similar fever in monkeys. It seemed to be conveyed by aerial infection, and no drug was of specific effect in the treatment. Thin and Manson denied that it was connected with malaria any more than with typhoid; but it occurs in China and other parts of the world as well as in Malta, and appears to be spreading over an increasing area. The mortality does not exceed two per cent., but patients are in hospital for an average of ninety days, and the prevalence of the disease is an especially serious matter where large numbers of troops have to be quartered.

[1] Med. Rec., Mar. 21. [2] Med. Week, July 3. [3] Med. Week, July 10. [4] B. M. J., May 16. [5] Ed. Med. Jour., May. [6] Am. Jour. Med. Sciences, March. [7] B. Med. Jour., May 2. [8] Glasgow Med. Jour., May. [9] B. M. J., May 30. [10] B. M. J., May 30. [11] Therap. Gaz., Feb. 15. [12] B. M. J., June 6. [13] Therap. Gaz., June. [14] Les Nouv Remédes, May. [15] B. M. J., March 21, and Birmingham Med. Rev. [16] B M. J., Ap. 4. [17] Practit., June. [18] Lancet March 14. [19] Amer. M.S. Bulletin, March 14. [20] Med. Chron., April. [21] B. M. J., March 21. [22] B. M. J., May 16. [23] N. Y. Med. Jour., June 6. [24] N. Y. Med. Jour., June 27. [25] Lancet, March 28. [26] Therap. Gazette, Feb. 16. [27] Med. Record, Ap. 11. [28] Med. Rec. Mar. 21.

